# Craniofacial Malformations as Fundamental Diagnostic Tools in Syndromic Entities

**DOI:** 10.3390/diagnostics12102375

**Published:** 2022-09-30

**Authors:** Ali Al Kaissi, Sergey Ryabykh, Nabil Nassib, Sami Bouchoucha, Lamia Benjemaa, Imen Rejeb, Syrine Hizem, Vladimir Kenis, Franz Grill, Susanne Gerit Kircher, Mohammad Shboul, Farid Ben Chehida

**Affiliations:** 1 National Medical Research Center for Traumatology and Orthopedics n.a. G.A. Ilizarov, 640032 Kurgan, Russia; 2 Department of Paediatric Orthopedics, Children Hospital, Tunis 1029, Tunisia; 3 Department of Human Genetics, Mongi Slim Hospital, Tunis 2046, Tunisia; 4 Pediatric Orthopedic Institute n.a. H. Turner, Department of Foot and Ankle Surgery, Neuroorthopaedics and Systemic Disorders, 196605 Saint-Petersburg, Russia; 5 Orthopedic Hospital of Speising, Pediatric Department, 1130 Vienna, Austria; 6 Department of Medical Patho-Chemistry and Genetics, Medical University of Vienna, 1090 Vienna, Austria; 7 Department of Medical Laboratory Sciences, Jordan University of Science and Technology, Irbid 22110, Jordan; 8 Ibn Zohr Institute of Radiology and Imaging, Tunis 1003, Tunisia

**Keywords:** craniofacial malformation, Idaho syndrome, Russel–Silver syndrome, contractual arachnodactyly Beals, Parry–Romberg syndrome

## Abstract

**Background:** A long list of syndromic entities can be diagnosed immediately through scrutinizing the clinical phenotype of the craniofacial features. The latter should be assisted via proper radiological interpretations. **Patients and Methods:** Different children aged from 1 month to 12 years were referred to our departments seeking orthopedic advice. Primarily, all received variable false diagnoses in other institutes. Two unrelated boys of one month and 12 months were falsely diagnosed as having positional plagiocephaly associated with contractures of idiopathic origin. Two unrelated boys of 14 months and 2 years were diagnosed with pseudo-hydrocephalus and non-specific syndrome, and were referred to explore their skeletal development. Two unrelated girls of 4 years old and 12 years old presented with multiple contractures were referred because of progressive scoliosis. A 4-year-old girl was referred with a false provisional diagnosis of facial diplegia. All children underwent detailed clinical, radiological and tomographic phenotypic characterizations and genetic testing, respectively. **Results:** Idaho syndrome (craniosynostosis associated with multiple dislocations) was the final diagnosis in the two unrelated boys with plagiocephaly and multiple contractures. Two children falsely diagnosed with pseudo-hydrocephalus and non-specific syndrome, were diagnosed with Silver–Russell syndrome (RSS). Contractural arachnodactyly Beals (CAB) was confirmed as the definitive diagnosis in the two unrelated girls with progressive scoliosis and multiple contractures. Parry–Romberg syndrome (PRS) associated with congenital lumbar kyphosis was the final diagnosis of the girl with the diagnosis of facial diplegia. Hypomethylation of ICR1 was confirmed in the RSS patients. Whole exome sequencing (WES) revealed a heterozygous mutation in the PRS patients. WES and array-CGH showed that no relevant variants or copy number variations (CNV) were identified in the CAB patients. **Conclusions:** On the one hand, newborn children can manifest diverse forms of abnormal craniofacial features, which are usually associated with either major or minor dysmorphic stigmata. A cleft lip/ palate is a major craniofacial malformation, and frontal bossing or a disproportionate craniofacial contour can be falsely considered as a transient plagiocephaly, which is spontaneously resolved by time. On the other hand, many physicians fall into the problem of deeming a countless number of diseases, such as contractures, as an idiopathic or non-specific syndrome. The latter stems from limited clinical experience. Therefore, failing to establish between the onset of the deformity and other inexplicit abnormal features that the patient or their immediate families or relatives carry is the final outcome. In this study, we used, for the first time, a reconstruction CT scan to further delineate the congenital disruption of the craniofacial anatomy and the other skeletal malformation complex.

## 1. Introduction

Infants with Idaho syndrome usually exhibit a dysmorphic craniofacial contour associated with multiple contractures. The clinical phenotype of the skull looks elongated from front to back because of the early closure of the metopic and the sagittal suture, which ends up with scaphocephaly. Limb deformities consist of multiple contractures due to a noticeable complete anterior dislocation of the tibia and fibula with absent patellae. The thumbs are abnormally implanted and there are contractures along the interphalangeal joints, which give rise to camptodactyly [1,2]. 

Children with Silver–Russell syndrome (RSS) usually manifest specific craniofacial features, prenatal growth deficiency and hemi hypertrophy of the limbs, which are the main features of this condition. The head phenotype is somehow confusing, despite the head circumference being relatively normal, and many pediatricians misdiagnose it as hydrocephalus. In fact, it can be termed as ‘pseudohydrocephalus’ [3,4,5,6]. 

The contractual arachnodactyly of Beals (CCB) syndrome has been a topic of confusion within the dysmorphology literature. It was first confused with Marfan’s syndrome, which is a specific and totally separate entity. Contractual arachnodactyly (CA) is characterized by joint contractures, present at birth, involving the fingers, knees, hips and elbows to a variable extent. The fingers are long and, radiologically, there is a considerable elongation of the proximal phalanges of the digits. The helix is flattened and crumpled, with some loss of the architecture. The most serious complication is that of scoliosis [7,8,9].

Parry–Romberg syndrome (PRS) (OMIM 141300) contralateral Jacksonian epilepsy can be encountered. PRS is characterized by a unilateral atrophy of bone and soft tissue, a midline facial groove and, sometimes, trigeminal neuralgia and contralateral Jacksonian epilepsy [10,11,12].

The value of presenting this paper is three-fold; Firstly, it is to prove that clinical and radiological phenotypic characterizations are the baseline tools used to approach a definite diagnosis; Secondly, etiology understanding is crucial for successful management; Thirdly, it is to demonstrate that the genotype is not a precise index for the assessment of the severity and the natural history of the phenotype.

## 2. Materials and Methods

The study protocol was approved by Ethics Committee of the (Ilizarov Scientific Research Institute, No.4 (50)/13.12.2016 Kurgan, Russia. Informed consent was obtained from the patient’s guardians. This study was conducted by the first author through his work at orthopedic Hospital of Speising, Vienna and his research partnership as a visiting Prof. to Ilizarov Scientific Research Institute and Turner Institute-Saint Petersburg, Russia and department of pediatric orthopedics, children Hospital (Becher-Hamza), Tunis.

The diagnostic process started through comprehensive recording of every stage of fetal life, from conception to delivery. The antenatal, prenatal, perinatal and postnatal events are sensitive topics requiring proper coverage. Therefore, the antenatal record of every child was explored carefully, and included maternal history of multiple spontaneous miscarriages (this is defined as three consecutive gestational losses prior to 20 weeks). Families with a history of multiple spontaneous fetal loss underwent a series of investigations (chromosomal analysis of parents, anticardiolipin antibody IgG, and IgM, hormonal assessment of TSH, homocysteine, factor V Leiden (a mutation of one of the clotting factors in the blood), prothrombin promoter mutation, activated protein C resistance (activated protein C resistance is a common cause of thrombophilia)). We revised the hysterosalpingography records of all of the mothers. No history of congenital uterine malformation, adhesions or any uterine anatomic malformation was evident. For instance, history of multiple spontaneous miscarriages/abortions, still births and neonatal mortalities have been recorded in almost every family, but with variable intensity. Sadly, none of the lost fetuses underwent chromosomal and or radiological investigations. We categorized every single patient in accordance with their craniofacial diagnostic features in combination with other musculoskeletal abnormalities. Therefore, clinical examination was set up primarily via eliciting any unusual signs of the craniofacial contour or any abnormal skin stigmata. Growth was assessed from pre and postnatal perspectives. Musculo skeletal examination was carried out to assess mobility of the joints (hyperlax or stiff), limb proportions, hands and foot plus spine phenotype. Neurological, hearing, vision and cardiorespiratory assessment are mandatory to exclude or confirm any visceral involvement, such as hepato-splenomegaly. Searching for relevant skeletal malformations required us to assess the clinical phenotype of up to three family generations. We usually asked for photos, radiographs, MRI and CT if they were available. Skeletal survey was carried out on every child presented with malformation complex (based on lateral skull, AP spine, AP pelvis and AP hand radiographs). Laboratory investigations, consisting of chromosomal karyotyping, whole exome sequencing (WES) and FISH test, were applied to all children. Interestingly, in all of these families, we experienced subjects either from maternal or paternal side with minimal malformations/ deformities and some with intellectual disabilities that, in fact, were strongly correlated to the diagnosis. We further subdivided this group of patients into their final definitive diagnosis. Investigations for urinary glycosaminoglycans (GAGs) were required in suspicious cases akin to lysosomal storage disorders.

## 3. Results

### 3.1. Idaho Syndrome (Craniosynostosis with Multiple Dislocations)

**Figure 1:**
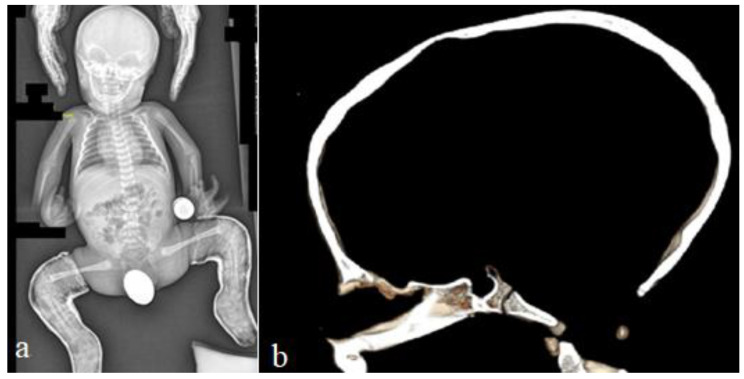
Skeletal survey; general skeletal radiograph at the age of one month showed multiple malformation complexes, starting from the skull and extending downwards to involve the upper and lower limbs, respectively. The skull showed apparent synostosis of the metopic suture. The upper limbs and lower limbs were notably associated with multiple dislocations (hips, wrists, knees, ankle and camptodactyly at the interphalangeal joints). The limb abnormalities were diagnostically important, in that there was a complete anterior dislocation of the tibia and fibula. Treatment was carried out using a plaster cast fixation of the hyperextended knee dislocation and clubfoot, due to the manipulation of the surgeon who, with one hand, held the knee in flexed position, shifting the tibia anterior and pushing the foot into the corrective position (**a**). 3D sagittal cranial CT scan showed craniosynostosis of the metopic suture associated with a well- formed ridge spreading from the metopic suture to involve entire sagittal suture, associated with the obliteration of the anterior fontanelle, with the eventual development of scaphocephaly (**b**).

**Figure 2:**
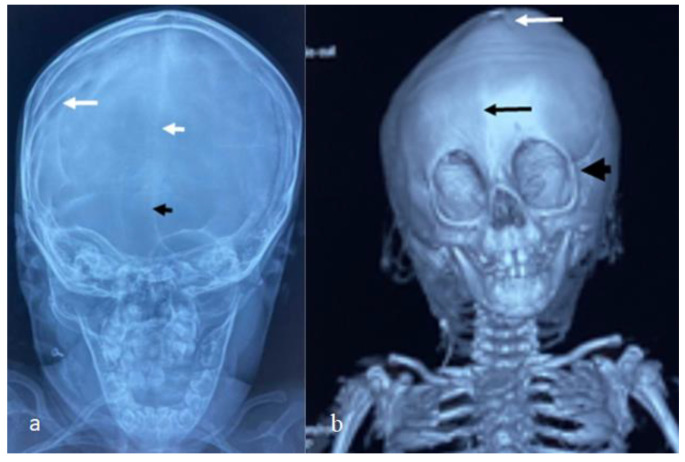
AP skull radiograph at the age of 12 months-old-boy- showed early closure of the metopic (black arrow head) and sagittal sutures (white arrow head) and partial closure of the right coronal suture (white arrow), leading to development of skull–crown asymmetry. Three-dimensional reconstruction CT scan of the same child at the age of 14 months showed a well-defined palpable bony ridge (black arrow) extending from the metopic suture upwards to include the sagittal suture (white arrow) (**a**); Note that the extremely large orbital cavities (arrowhead) with diffuse early closure of the metopic and the sagittal sutures arrow), leading to the development of a dysmorphic and asymmetrical contour of the head because of the unilateral upward bulging of the crown (white arrow). Asymmetrical contour of the cranium resulted from the unilateral partial closure of the right coronal suture with the simultaneously but asymmetrical early closure of the squamosal sutures (**b**).

**Figure 3:**
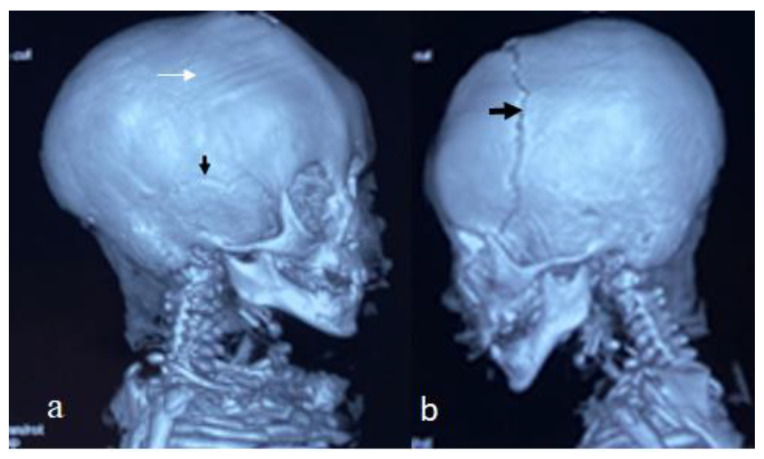
Three-dimensional reconstruction CT scan showed the disproportion in growth between the cranial and facial bones. Apparent closure of the metopic and sagittal sutures led to the development of scaphocephaly. Interestingly, there was a unilateral early closure of the right coronal (white arrow) and early closure of the squamosal sutures, respectively (black arrow), causing the asymmetrical bulging of the central part of the crown (**a**). Three-dimensional reconstruction confirmed the persistence of the left coronal suture (arrow) (**b**).

For this syndrome, we clinically investigated two unrelated affected boys. The first was a one-month-old boy presenting with multiple contractures who was falsely diagnosed in other institutes as having positional plagiocephaly associated with idiopathic multiple contractures. A clinical examination at the age of one month showed growth deficiency (-2SD) and OFC (around the 75th percentile). Clinically, he manifested an abnormal craniofacial contour with prominent sloping of the frontal area. The helices were over-folded. The palpation of the skull gives the impression of a newly formed ridge along the metopic suture and a newly formed ridge along the sagittal suture, with a feeling of a rudimentary anterior fontanelle (the skull is elongated from front to back). The musculo-skeletal examination showed ligamentous hyper laxity but not hypotonia. Multiple contractures over the upper and lower limbs were, respectively, associated with multiple dislocations (hips and knee joints). Skeletal survey: a general skeletal radiograph at the age of one month showed multiple malformation complex, starting from the skull and extending downwards to involve the upper and lower limbs, respectively. The skull showed an apparent synostosis of the metopic suture. The upper limbs and lower limbs were notably associated with multiple dislocations (hips, wrists, knees and ankle, in addition to camptodactyly at the interphalangeal joints). The limb abnormalities were diagnostically important, in that there was a complete anterior dislocation of the tibia and fibula, and the patellae were absent (Figure 1a). A sagittal cranial CT scan showed craniosynostosis of the metopic suture associated with a well- formed ridge spreading from the metopic suture to involve the anterior portion and the posterior part of the sagittal suture, associated with the obliteration of the anterior fontanelle, with the eventual development of scaphocephaly (Figure 1b). The second child is a 12-month-old-boy- presenting with multiple dislocations on top of a malformative craniosynostosis. He manifested apparent craniofacial dysmorphic features (facial asymmetry, proptosis, depressed nasal bridge, long philtrum and a low set malformed ears). An AP skull radiograph at the age of 12 months showed an early closure of the metopic (black arrow head) and sagittal sutures (white arrow head) and partial closure of the right coronal suture (white arrow), leading to the development of skull–crown asymmetry. A 3D reconstruction CT scan of the same child at the age of 14 months showed a well-defined palpable bony ridge (black arrow) extending from the metopic suture upwards to include the sagittal suture (white arrow) (Figure 2a). Note that the extremely large orbital cavities (arrowhead) with diffuse early closure of the metopic and the sagittal sutures (arrow), leading to the development of a dysmorphic and asymmetrical contour of the head because of the unilateral upward bulging of the crown (white arrow). Asymmetrical contour of the cranium resulted from the unilateral partial closure of the right coronal suture and simultaneous but asymmetrical early closure of the squamosal sutures (Figure 2b). A 3D reconstruction CT scan showed the disproportion in growth between the cranial and facial bones. The apparent closure of the metopic and sagittal sutures led to the development of scaphocephaly. Interestingly, there was a unilateral early closure of the right coronal (white arrow) and early closure of the squamosal sutures, respectively (black arrow), causing the development of asymmetrical bulging of the central part of the crown (Figure 3a). A 3D reconstruction confirmed the persistence of the left coronal suture (arrow—Figure 3b). Chromosomal karyotyping and the FISH test were normal, and there were no disease-causing copy number variations (CNV) using array-CGH analysis.

### 3.2. Silver–Russel Syndrome: Pre-and Postnatal Growth Retardation and a Confusing Large Head Associated with Skeletal Asymmetry

Two unrelated boys (14 months old and 2 years old, respectively) presented with RSS, and craniofacial features were the first to be recognized and facilitate the definitive diagnosis.

**Figure 4:**
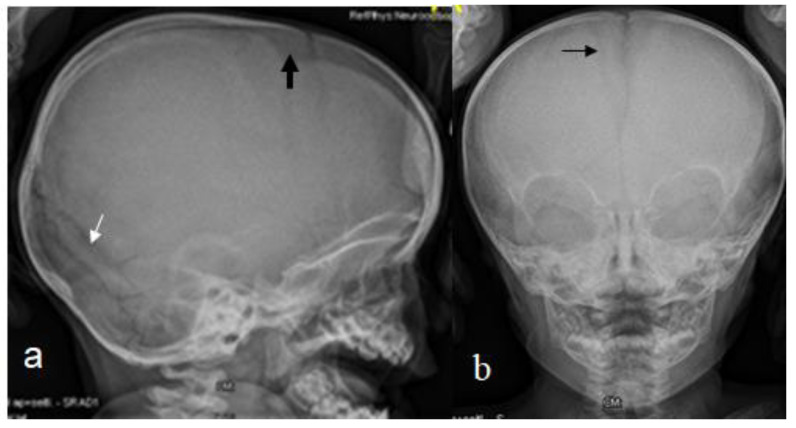
Lateral skull radiograph of a 14-month-old boy with RSS showed a very large anterior fontanelle (massive defective ossification) associated with frontal bossing, giving the impression of pseudo-hydrocephalus-arrow hyperostosis of the skull base and Wormian bones along the lambdoid sutures (**a**). AP skull radiograph showed the frontal bossing and the persistence of the anterior fontanelle-arrow (**b**).

**Figure 5:**
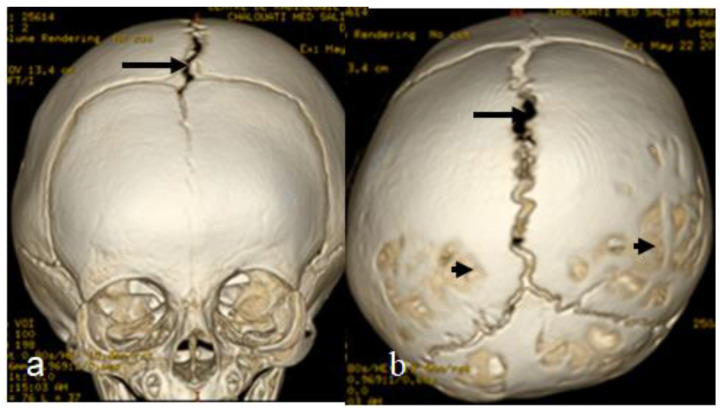
Three-dimensional reconstruction CT scan of the skull (anterior view) of a 2-year-old boy with RSS showed the frontal bossing and the persistent open anterior fontanelle-(arrow) (**a**); Three-dimensional reconstruction of the crown of the skull of the same boy showed defective ossification of the anterior fontanelle and defective and disorganized ossification of the skull bones (arrow heads) (**b**).

The 14-months-old boy was referred to our consultation for a clinical assessment and diagnosis. The clinical examination revealed a growth deficiency of -2SD, pseudo-hydrocephalus with a prominent forehead, deep seated eyes and sparse hair. Down slanting palpebral fissures, a beaked nose with a protruding nasal septum below the hypoplastic nares, low set ears and thin lips were notable features. The thin upper lip was associated with retrognathia. The limbs were short, the thumbs were relatively short and wide, the hallux was wide and the rest of the digits of the hands and feet were short. Relative hemi-hypertrophy could be elicited. The hands were with relative camptodactyly. A musculoskeletal examination showed moderate ligamentous hyperlaxity. The intellectual performance was difficult to assess, though his smile was somehow associated with peculiar facial expressions overwhelmed by a bizarre facial grimacing when trying to smile. The skeletal survey and lateral skull radiograph showed a very large anterior fontanelle (massive defective ossification) associated with frontal bossing, giving the impression of pseudo-hydrocephalus. There were also hyperostosis of the skull base and Wormian bones along the lambdoid sutures (Figure 4a). An AP skull radiograph showed the frontal bossing and the persistence of the anterior fontanelle (Figure 4b). Genetic testing was not performed for this patient. A 2-year-old boy presented with the phenotype and genotype of RSS. He underwent a 3D reconstruction CT scan to assess the anatomy of the skull and the anterior fontanelle. A 3D reconstruction CT scan of the skull (anterior view) of the 2-year-old boy showed frontal bossing and the persistent open anterior fontanelle (Figure 5a). A 3D reconstruction of the crown of the skull of the same boy showed a defective ossification of the anterior fontanelle and a defective and disorganized ossification of the skull bones (arrows) (Figure 5b). Hypomethylation of the imprinting control region (ICR1) on chromosome 11p15 and maternal uniparental disomy of chromosome 7 was encountered.

### 3.3. Contractual Arachnodactyly Beals Syndrome (CAB)

**Figure 6:**
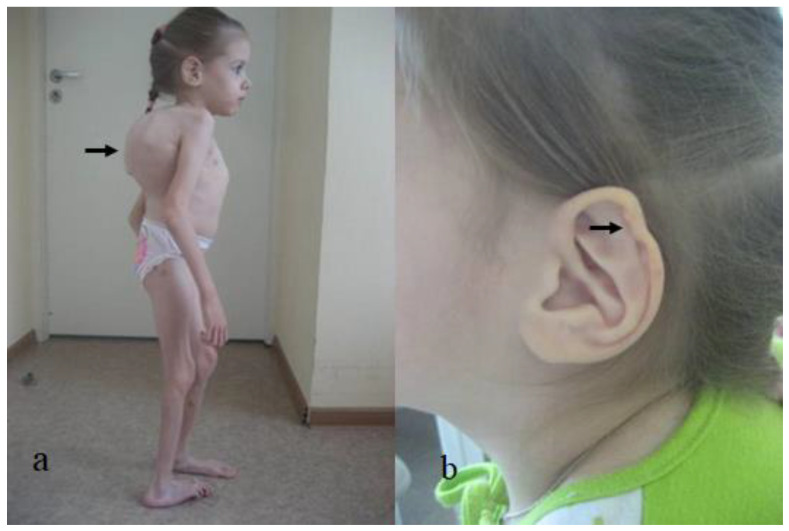
Clinical phenotype of a 4-year-old girl showed marfanoid habitus, a long face with a large and wide frontal area, hypertelorism, marfanoid-like features (thin and long slender limbs), kyphoscoliosis (arrow), long fingers (arachnodactyly) and apparent bilateral pes equinovalgus. The head was brachycephalic, with a long philtrum and a high vault palatine (**a**); The ears showed specific abnormalities (helix was flattened and crumpled, with some loss of the architecture (arrow)) (**b**).

**Figure 7:**
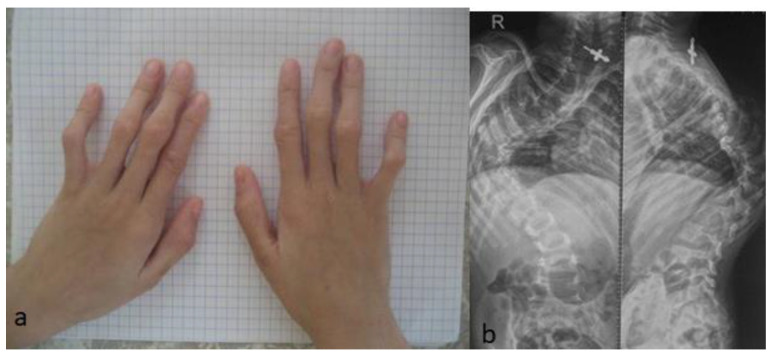
Arachnodactyly associated with camptodactyly of the 5th. Fingers, in a 4-year-old girl (**a**); AP and lateral spine radiographs showed kyphoscoliosis (Cobbs angle of 80°) (**b**).

**Figure 8:**
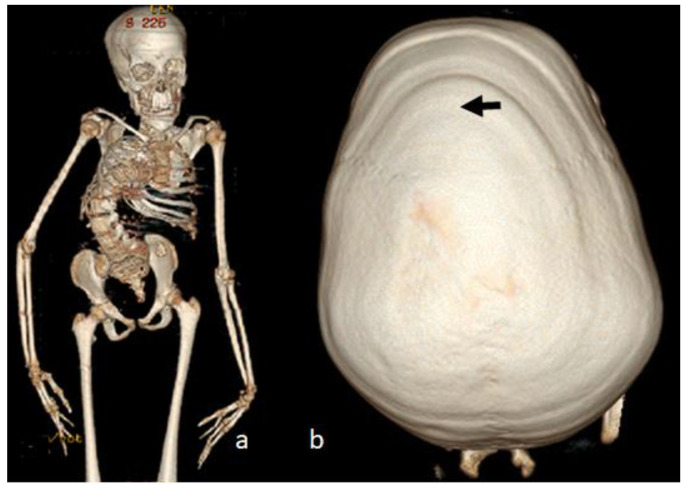
A 12-year-old girl was referred because of scoliosis associated with multiple contractures. Three-dimensional phenotype reconstruction was applied to explore the skeletal system in full details (**a**). She manifested the full clinical criteria of Marfan-like syndrome (**a**). Long, thin limbs, arachnodactyly, multiple contractures and principally progressive kyphoscoliosis, with a Cobbs angle of 90° (**a**). Three-dimensional reconstruction carried out to explore the crown of the skull showed asymmetrical development associated with closure of the sagittal suture (arrow) (**b**).

Two unrelated girls (4 years old and 12 years old) presented with multiple contractures. The 4-year-old girl presented with specific craniofacial features, marfanoid habitus and arachnodactyly. She was born full term as a product of non-complicated gestation. At birth, she manifested a crumpled irregular superior helix associated with multiple joint contractures, involving the fingers, knees, hips and elbows, and scoliosis. Her subsequent course of development was characterized as being awkward. The clinical phenotype of a 4-year-old-girl showed marfanoid habitus, a long face with a large and wide frontal area, hypertelorism, marfanoid-like features (thin and long slender limbs), kyphoscoliosis (arrow), long fingers (arachnodactyly) and apparent bilateral pes equinovalgus. The head was brachycephalic, with a long philtrum and a high vault palatine (Figure 6a). The ears showed specific abnormalities (the helix was flattened and crumpled, with some loss of the architecture (arrow)) (Figure 6b). A photo of the hands showed arachnodactyly and camptodactyly of the 5th fingers (Figure 7a). AP and lateral spine radiographs showed kyphoscoliosis (Cobbs angle of 80°) (Figure 7b). The 12-year-old girl was referred because of progressive scoliosis associated with multiple contractures. Three-dimensional phenotype reconstruction was applied to explore the skeletal system in full detail. She manifested the full clinical criteria of Marfan-like syndrome: long, thin limbs, arachnodactyly, multiple contractures and principally kyphoscoliosis, with a Cobbs angle of 90° (Figure 8a). A 3D reconstruction carried out to explore the crown of the skull showed asymmetrical development associated with closure of the sagittal suture (arrow), while the posterior part was somehow still preserved (arrow) (Figure 8b). Whole exome sequencing revealed a heterozygous mutation in the *FBN1* gene (OMIM 134797) in both patients.

### 3.4. Parry–Romberg Disease (Birth Defects Possibly in Correlation with Pre-Gestational Diabetes)

**Figure 9:**
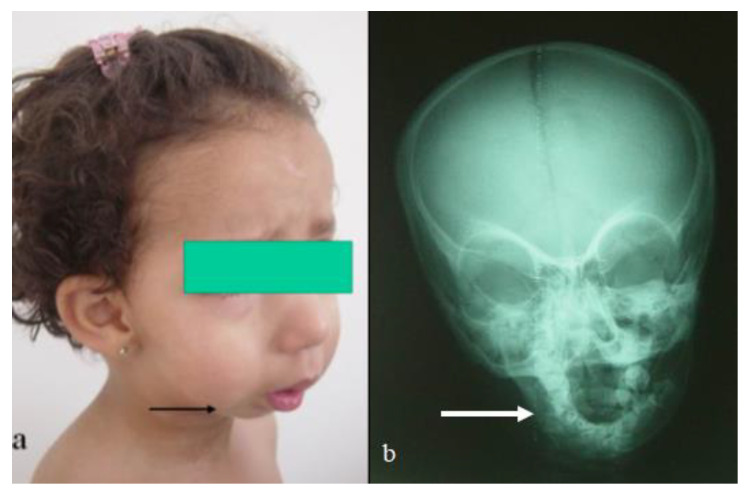
Clinical photo of a 4-year-old- girl with progressive hemi-atrophy of the right side of the face, which shows apparent unilateral facial atrophy of the right side of the face (arrow) (**a**); AP skull radiograph showed apparent atrophy of the right side of the mandible, giving rise to unilateral micrognathia (right sided atrophy of the maxilla, and the alveolar process of the maxilla) (arrow) (**b**).

**Figure 10:**
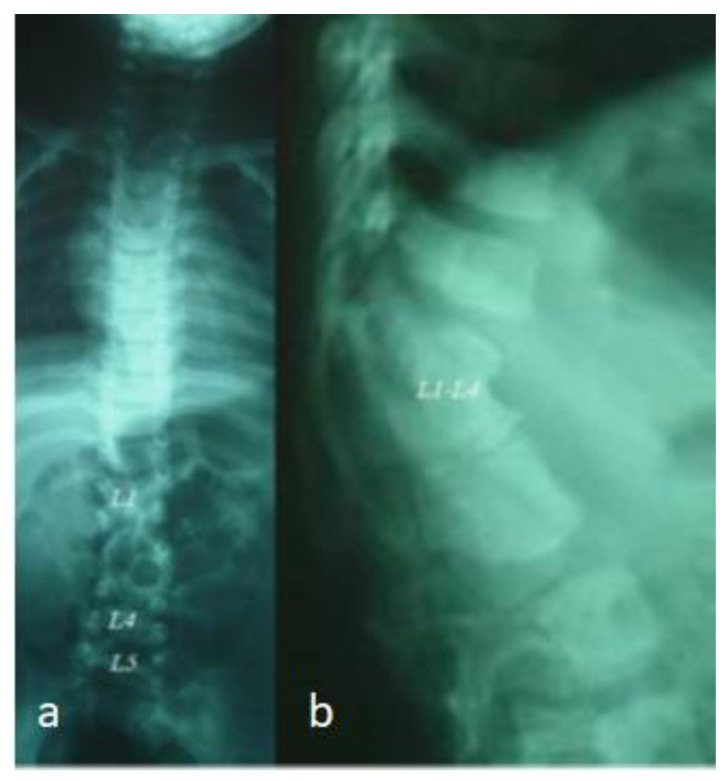
AP spine radiograph showed diffuse malsegmentation of L1–4 and spina bifida occulta of L5 (**a**); Lateral spine radiograph showed progressive lumbar kyphosis (**b**).

A 4-year-old girl presented with progressive unilateral left sided facial atrophy (fronto-maxillary defects), which was falsely diagnosed as facial diplegia. She was born full term as a product of a fluctuating non-controllable maternal diabetes. Her subsequent course of development was with normal parameters. After the age of three and a half years, her parents observed a progressive symmetry of the face. The mother said that her daughter had an unusual rise in temperature for one week, followed by a gradual and slowly progressive loss of subcutaneous fat and muscle of the right side of the face, and she started to complain of cluster headaches (Figure 9a). There was no history of convulsions or any neurological deficits. The family history showed migraine as a uniform complaint in her mother and all aunts and uncles, and a ruptured cerebral aneurysm was the reason for the death of a young cousin. Interestingly, uncontrolled fluctuating pregestational diabetes mellitus was identified. A clinical examination showed a growth deficiency and unilateral facial atrophy of the right side of the face (progressive weakness and atrophy of the masticatory muscles, associated with a noticeable retardation of ipsilateral tooth eruption). An examination of the buccal cavity revealed right sided tongue atrophy. Palpation of the jaw showed apparent hypoplasia. A musculo-skeletal examination showed moderate ligamentous hyperlaxity and small hands and feet. Interestingly, lumbar kyphosis was evident. Skeletal survey: the AP skull radiograph showed apparent atrophy of the right side of the mandible, giving rise to micrognathia (right sided atrophy of the maxilla, and the alveolar process of the maxilla) (Figure 9b). An AP spine radiograph showed diffuse malsegmentation of L1–4 and spina bifida occulta of L5 (Figure 10a). A lateral spine radiograph showed progressive lumbar kyphosis (Figure 10b). Whole exome sequencing and the FISH test did not reveal positive results.

## 4. Discussion

An early diagnosis is the key factor in clinical medicine. The process of diagnosis requires prompt and deep observational insights from the clinician. The responsibility of the clinician is strongly correlated to their reading capability and interpretive potential through a careful analysis of every unusual clinical sign. The commencement of history and the family gathering of information can be meaningful and fruitful when connected to the current deformity. A detailed clinical examination can facilitate the clinician’s path towards the finalization of the diagnostic dilemma. The importance of a definitive and distinct diagnosis lies in protecting the children and their parents from the psychological trauma and agony, which are of common occurrence, especially when the child falls in the hands of a physician with limited knowledge and experience [13,14,15]

Surgical intervention for craniosynostosis should be the first line of treatment performed in children presenting with Idaho syndrome. The purpose is to halt the progressive course of synostosis in order to regain the normalcy of the physiological intracranial dynamics and to re-maintain the normal craniofacial growth [1,16]. Silver–Russell syndrome (RSS) is a clinically heterogeneous disorder that shows a characteristic facial appearance, pre and post-natal growth retardation and asymmetry of the limbs. The maternal duplication of 11p15 might be responsible for severe intrauterine and postnatal growth retardation [17]. The congenital contractual arachnodactyly of Beals (CCB) is an autosomal dominant connective tissue disorder characterized by contractures, long fingers, and, radiologically, a considerable elongation of the proximal phalanges of the digits (arachnodactyly) [18]. Parry–Romberg syndrome is characterized by unilateral atrophy of bone and soft tissue, a midline facial groove and, sometimes, trigeminal neuralgia and contralateral Jacksonian epilepsy [11]. Gestational diabetes is a notoriously unpredicted ailment for the growing fetus. A multisystem can be pathologically involved (neurogenic, osteogenic and cardiovascular) [19].

The value of presenting this paper is threefold; firstly, it is to prove that clinical and radiological phenotypic characterizations are the baseline tools used to approach a definite diagnosis. A correct and precise reading of the clinical signs plays the major role towards achieving the diagnostic process. Similarly, misinterpretations of vital radiographic stigmata can leads to a significant decline in detecting the etiology understanding, especially in patients with long-term ill-defined skeletal deformities. The number of physicians that are indulged in daily practice and capable of performing a detailed clinical examination, but unfortunately failing to identify and read the signs that they see, is accelerating.

Secondly, etiology understanding is crucial for successful management. Much of our clinical work is centered on one simple rule that every skeletal deformity/abnormality must have an underlying causality that needs to be explored and addressed. This stems from the conviction that the vast majority of the skeletal deformities—if not all—do not occur randomly. Many physicians fall into the problem of deeming a countless number of diseases idiopathic, as no clear connection has been established between the onset of the deformity and other inexplicit abnormal features that the patient or their immediate families or relatives carry. Our clinical potential focuses on linking these connections and reiterating the fundamental rule that etiological understanding is paramount to successful management and treatment.

Thirdly, it is to demonstrate that the genotype is not a precise index for the assessment of the severity and natural history of the phenotype. We believe that the vast majority of patients with a skeletal malformation complex represent a puzzle that requires thorough knowledge and experience, and, certainly, the genotype is a required tool for genetic counselling.

## 5. Conclusions

The congenital distortion of the craniofacial anatomical structures can be of great assistance to orthopedic physicians via the recognition of specific clinical stigmata that fall outside the normal course of human development. Genuine clinical efforts should be directed towards fundamental rules, where every congenital or late-onset skeletal deformity /abnormality must have an underlying causality that needs to be explored and addressed. This stems from the conviction that the vast majority of skeletal deformities—if not all—do not occur randomly. My clinical experience focuses on uncovering and emphasizing these connections and reiterating the fundamental rule that etiological understanding is paramount to successful management and treatment. Successful clinical/radiological readings provide impactful guidance towards comprehensive orthopedic management.
